# The Man and the Moment

**Published:** 1916-04-15

**Authors:** 


### The Hospital

The Workers' Newspaper of Administrative Medicine and Institutional Life, Administration, National Insurance and Health.

No. 1557, Vol. LX. SATURDAY, APRIL 15, 1916. PRICE ONE PENNY

### THE MAN AND THE MOMENT.

There has been much in the lay Press in recent months which has led to discussion as to whether or no the United Kingdom and the Empire has within its borders at the present time a man or men of the moment. In the hospital world one striking example of the man and the moment is the position of deserved influence and authority which the Honourable Arthur Stanley has attained in it since the beginning of the present year. For thirty years, that is, ever since an increasing number of hospitals began to recognise the importance of selecting and training nurses on a definite plan, there has been an increasing desire, quite apart from individual prejudice in favour of this or that system of training, to unite the whole body of attendants on the sick and to give them a standard training, a uniformity of certificate through one portal, more adequate remuneration, and complete protection from the abuse of their uniform and the evils arising from the competition of relatively incompetent or imperfectly trained women. In its issue of January 1, 1916, the *Nursing Mirror* in a forcible article set forth the chaotic condition of the nursing world in these matters, and insisted upon the paramount necessity for united action on the part of the great nurse-training schools, as well as of certificated trained nurses themselves. It was pointed out that any objection to such immediate action based upon the claim that war time was inopportune for such a movement had no substantial ground to rest on, seeing that unless immediate steps were taken to unite the whole body of trained nurses, and those who were responsible for their training and education, the end of the war might find the country, so far as nursing was concerned, in a condition of chaos, which must prove lamentable and even dangerous. This article was written by one who has kept the hospital field under continuous observation for fifty years, and the soundness of the view expressed has been completely demonstrated by the events which have followed its publication.

The occasion immediately produced the man in the person of the Hon. Arthur Stanley, M.P., who as the Chairman of the Joint War Committee of the British Red Cross Society and the Order of St. John, has rendered invaluable service as an organiser and man of affairs. Mr. Stanley had been impressed with the need for action in the matters referred to. He took counsel with a number of advisers, he invited the co-operation of those who had technical knowledge and influence in the hospital world, and formulated a plan which has now taken shape as the College of Nursing, a company limited by guarantee and not having a share capital. In successive numbers we have given full information of the steps whereby this result has been achieved, and of the fact that it has succeeded in uniting not only, practically, all the matrons of important hospitals and superintendents of nurses, who are at present engaged in the active practice of their profession, but the hospital managers throughout the country, and, with the doubtful exception of a few who are waiting on events, the overwhelming majority of sisters and nurses at present actively pursuing their profession.

This success was demonstrated in a remarkable way at the meeting which Mr. Arthur Stanley summoned to assemble in the Governors' Hall of St. Thomas's Hospital on the afternoon of April 7. We publish a verbatim report this week of the proceedings, for they must prove of historic value. It was altogether a most remarkable and representative gathering of hospital managers, matrons, superintendents, sisters, and nurses from many parts of the United Kingdom. Mr. Arthur Stanley was in the chair, and his conduct of the meeting won the confidence of everyone present, with results which cannot fail to be momentous for good to the whole profession of nursing and to all who realise the value of trained and competent service for the suffering and the sick. A life's experience and a full recognition of the underlying difficulties of the task which lay before Mr. Stanley before the meeting commenced, make the admiration of the success which he won from his audience overwhelming. It was not merely that all present felt themselves indebted to the Chairman, but that the feeling of confidence was probably deepest and most complete in those men and women present who had the most intimate knowledge of the history of the development of nursing in these islands during the last forty, and especially during the last thirty, years. This was deepened by a complete apprehension of the actual difficulties and dangers which had threatened both the hospitals and the nurses previous to the incorporation of the College of Nursing.

Here undoubtedly was the man of the moment, a fact which everyone gratefully recognised, and to that fact the future development and immense power for good of the College of Nursing may fitly be credited. Mr. Arthur Stanley has had, as he very properly acknowledged, the help of some of—we had nearly said of all—the ablest minds available in the nursing field, for we are confident that there is not one single disinterested well-wisher of British nurses and British nursing who is not in favour of the establishment of a College of Nursing. Is there, indeed, one who does not look with gratitude to its founders for the unity, security, and direct representation of trained nurses everywhere which its incorporation and its Representative Council, a majority of whom have already been appointed, convincingly promises?

We hope by the time the war is over Mr. Stanley may have gained, like so many other men, so keen an interest in our hospitals, and the splendid results achieved under the voluntary system, that he may be led to accept the treasurership of one of the oldest and greatest of these institutions.

